# Programmed death-1 blockade enhances the antitumor effects of peptide vaccine-induced peptide-specific cytotoxic T lymphocytes

**DOI:** 10.3892/ijo.2014.2737

**Published:** 2014-10-30

**Authors:** YU SAWADA, TOSHIAKI YOSHIKAWA, MANAMI SHIMOMURA, TATSUAKI IWAMA, ITARU ENDO, TETSUYA NAKATSURA

**Affiliations:** 1 Division of Cancer Immunotherapy, Exploratory Oncology Research and Clinical Trial Center, National Cancer Center, Kashiwa, Chiba 277-8577, Japan; 2 Department of Gastroenterological Surgery, Yokohama City University Graduate School of Medicine, Kanazawa-ku, Yokohama 236-0004, Japan

**Keywords:** programmed death-1, cytotoxic T lymphocyte, peptide vaccine, glypican-3, hepatocellular carcinoma

## Abstract

Novel treatment modalities are required urgently in patients with hepatocellular carcinoma (HCC). A vaccine that induces cytotoxic T lymphocytes (CTLs) is an ideal strategy for cancer, and glypican-3 (GPC3) is a potential option for HCC. Blocking the programmed death-1 (PD-1)/PD-L1 pathway is a rational strategy to overcome tumor escape and tolerance toward CTLs. In the present study, we investigated whether anti-PD-1 blocking antibodies (αPD-1 Ab) enhanced the number of vaccine-induced peptide-specific CTLs in peripheral blood mononuclear cells (PBMCs) following the administration of GPC3 peptide vaccine to both patients and in a mouse model. The inhibitory receptor PD-1 was highly expressed in *ex vivo* GPC3-specific CTLs isolated from the PBMCs of vaccinated HCC patients. *In vitro*, interferon-γ induced PD-L1 expression in liver cancer cell lines. In addition, PD-1 blockade increased the number of GPC3-specific CTLs, which degranulate against liver cancer cell lines. *In vivo* experiments using tumor-bearing mouse models showed that the combination therapy of peptide vaccine and αPD-1 Ab suppressed tumor growth synergistically. PD-1 blockade increased the number of peptide-specific tumor-infiltrating T cells (TILs) and decreased the expression of inhibitory receptors on TILs. This study demonstrated that PD-1/PD-L1 blockade augmented the antitumor effects of a peptide vaccine by increasing the immune response of vaccine-induced CTLs, and provided a foundation for the clinical development of a combination therapy using a GPC3 peptide vaccine and αPD-1 Ab.

## Introduction

Antigen-specific cancer immunotherapy using the induction of tumor-specific reactions without autoimmunity is a potentially attractive option for the treatment of cancer. However, immunotherapy for hepatocellular carcinoma (HCC) is still in the preclinical or early clinical trial phases (I and II) of development (1,2). Glypican-3 (GPC3), a carcinoembryonic antigen, is overexpressed in 72–81% of HCC cases, and is correlated with poor prognosis; therefore, it is an ideal target for HCC (3–7). Recently, a phase I clinical study of a GPC3-derived peptide vaccine reported its safety and efficacy for the treatment of advanced HCC (8). Although vaccine-induced GPC3-peptide-specific cytotoxic T lymphocytes (CTLs) are often tumor reactive *in vitro* (9) and correlate with overall survival, no complete response was observed when GPC3 peptide vaccination was used as monotherapy in patients with advanced HCC (8).

Programmed death-1 (PD-1) is expressed on activated T and B cells, and elicits inhibitory signals (10). Its ligand PD-L1 is member of the B7 family, and interacts with PD-1 (11). Several studies have shown that the PD-1/PD-L1 pathway plays a critical role in compromised tumor immunity (12,13). PD-1 antibody blockade exerts antitumor effects in clinical trials (14,15). High expression levels of PD-1 on T cells, both in tumor-infiltrating lymphocytes (TILs) and peripheral blood mononuclear cells (PBMCs), were correlated with poor prognosis in HCC patients after surgical resection (16). In addition, PD-L1 expression in HCC was correlated with tumor aggressiveness and postoperative recurrence (17).

In animal models, PD-1 blockade exerts synergistic effects with various tumor vaccines to enhance tumor antigen-specific T cell responses and suppress tumors *in vivo* (18–20). It was reported that melanoma vaccine-induced CTLs become exhausted, which could be reversed by blocking the inhibitory pathways (21). However, a study evaluating the combination of a cancer vaccine and an anti-PD-1 blocking antibody (αPD-1 Ab) for HCC has not been conducted. Therefore, the aim of this study was to investigate whether αPD-1 Ab would enhance the antitumor effects of a peptide vaccine by analyzing CTLs isolated from the PBMCs of vaccinated patients, as well as from a mouse model.

## Materials and methods

### Patient samples

Three clinical trials were conducted using GPC3-derived peptide vaccines. A phase I trial (n=33) was performed in patients with advanced or metastatic HCC (8) (University Hospital Medical Information Network Clinical Trials Registry; UMIN-CTR no. 000001395). Subsequently, a phase II trial was performed using a GPC3-derived peptide vaccine as an adjuvant therapy in patients with HCC (UMIN-CTR: 000002614, on-going). Finally, a pilot study of liver biopsies taken before and after GPC3 peptide vaccination is being performed for advanced HCC (UMIN-CTR: 000005093, on-going). These trials were approved by the Ethics Committee of the National Cancer Center, Japan, and conformed to the ethical guidelines of the 1975 Declaration of Helsinki. All patients were enrolled after providing written informed consent. Patients were injected intradermally with HLA-A24-restricted GPC3_298–306_ (EYILSLEEL) or HLA-A2-restricted GPC3_144–152_ (FVGEFFTDV) peptide vaccines emulsified with incomplete Freund’s adjuvant (IFA, Montanide ISA-51VG; SEPPIC).

Peripheral blood (30 ml) was obtained at the National Cancer Center Hospital East. PBMCs were isolated using standard Ficoll density gradient centrifugation from buffy coats. The remaining PBMCs were used after immunological monitoring in clinical trials. The immunological analyses were approved by the Ethics Committee of the National Cancer Center, Japan.

### Cell lines

The human liver cancer cell lines SK-Hep-1 (GPC3^−^, HLA-A^*^02:01/A^*^24:02), SK-Hep-1/GPC3 (GPC3^+^, HLA-A^*^02:01/A^*^24:02), and HepG2 (GPC3^+^, HLA-A^*^02:01/A^*^24:02) were available in our laboratory and were used as the target cells (6,9). SK-Hep-1/GPC3 is an established stable GPC3-expressing cell line that was transfected with the human GPC3 gene, whereas SK-Hep-1/vec is an established counterpart cell line that was transfected with an empty vector. The mouse lymphoma cell line RMA (OVA-, H-2K^b^) was provided by Dr Yasuharu Nishimura (Kumamoto University, Japan). Cells were cultured at 37°C in RPMI-1640 or DMEM (Sigma-Aldrich) supplemented with 10% fetal bovine serum (FBS), 100 U/ml penicillin and 100 μg/ml streptomycin in a humidified atmosphere containing 5% CO_2_.

### Synthetic peptides and cytokines

The peptides used in this study were as follows: HLA-A^*^02:01-restricted GPC3_144–152_ (FVGEFFTDV) peptide (American Peptide Co.), HLA-A^*^24: 02-restricted GPC3_298–306_ (EYILSLEEL) peptide (American Peptide Co.), HLA-A^*^02:01-restricted human immunodeficiency virus (HIV)_77–85_ (SLYNTYATL) peptide (ProImmune), and H-2K^b^-restricted ovalbumin (OVA)_257–264_ (SIINFEKL) peptide (AnaSpec). The peptides were dissolved and diluted in 7% NaHCO_3_ or dimethyl sulfoxide (DMSO). Where appropriate, liver cancer cell cultures were treated with 100 U/ml recombinant interferon (IFN)-γ (PeproTech).

### Ex vivo Dextramer staining and flow cytometry

PBMCs were stained using HLA-A^*^02:01 Dextramer-RPE [GPC3_144–152_ (FVGEFFTDV), HIV_19–27_ (TLNAWVKVV) or negative control; Immudex] and HLA-A^*^24:02 Dextramer-RPE [GPC3_298–306_ (EYILSLEEL), HIV_583–591_ (RYLKDQQLL); Immudex] for 15 min at room temperature, followed by anti-CD8-FITC (clone T8, Beckman Coulter), anti-PD-1-APC (clone EH12.2H7, BioLegend), or isotype control-APC (clone MOPC-21, BioLegend) for 20 min at 4°C. Flow cytometry was performed using a FACSCanto II (BD Biosciences).

### Blocking antibody

GPC3 peptide-specific CTL clones were established from PBMCs as described previously (9). The CTL clones were cultured in AIM-V medium (Life Technologies) supplemented with 10% human AB serum in the presence of 10 μg/ml anti-PD-1 (clone J116, eBioscience) or 10 μg/ml control (clone MOPC-21, BioXcell) monoclonal antibodies for 2 days.

### CD107a assay

GPC3 peptide-specific CTL clones were incubated with SK Hep-1/vec pulsed with GPC3_144–152_ or HIV_19–27_ peptide and HepG2 at a 1:1 ratio for 3.5 h at 37°C. CTL clones were stained with anti-CD107a-APC (clone LAMP-1, BD Bioscience) during the incubation period, followed by anti-CD8-FITC (clone LT8, ProImmune) for 20 min at 4°C.

### Mice

Female C57BL/6 mice (6–8 weeks old) were purchased from Japan Charles River Laboratories (Yokohama, Japan), and were maintained under specific pathogen-free conditions. The Animal Research Committee of the National Cancer Center, Japan, approved all studies. All animal procedures were performed according to the guidelines for the Animal Research Committee of the National Cancer Center, Japan. Ether was used for mouse euthanasia and anesthesia.

### In vivo tumor growth inhibition assays

It was reported previously that intratumoral (i.t.) injection of OVA_257–264_ peptide (SIINFEKL) effectively inhibited OVA-negative tumor growth and survival in a peptide vaccine model using C57BL/6 mice (22). RMA cells (1×10^5^ cells/100 μl PBS) were implanted on the backs of C57BL/6 mouse on day 0. They were then injected with 50-μg peptide mixed with an equal volume of incomplete Freund’s adjuvant (IFA, Montanide ISA-51VG; SEPPIC) on days 7 and 14. The total volume of injected vaccine solution was 100 μl in all experiments. For *in vivo* therapeutic experiments, anti-mouse PD-1 (clone 4H2) and control Ab (clone MOPC-21, BioXcell) were provided by Ono Pharmaceutical Co., Ltd. The anti-mouse PD-1 Ab (clone 4H2) used in the present study is a chimeric rat Ab containing the murine IgG1 Fc region (23). Anti-PD-1 or control Abs (200 μg/day) were injected intraperitoneally (i.p.) on days 7 and 14. Tumor volume was monitored twice per week, and was calculated using the following formula: tumor volume (mm^3^) = a × b × b × 0.5, where a is the longest diameter, b is the shortest diameter, and 0.5 is a constant to calculate the volume of an ellipsoid. Mouse health, behavior and mortality were checked daily. All mice were maintained until they showed signs of morbidity or the length or width of the tumors exceeded 30 mm, at which point they were sacrificed for reasons of animal welfare (22).

### IFN-γ enzyme-linked immunospot (ELISPOT) analysis

The BD^TM^ ELISPOT set (BD Biosciences) was used to assess the levels of IFN-γ, as described previously (24). Briefly, CD8-positive splenocytes (5×10^5^) were added to the plate as effector cells. Then, either bone marrow-derived dendritic cells (BM-DCs; 1×10^5^) pulsed with OVA peptide (10 μg/ml; as target cells) or non-pulsed BM-DCs (1×10^5^; as control cells) were added. The plate was then incubated for 37°C, for 20 h in the presence of 5% CO_2_. Spots were counted automatically using the Eliphoto system (Minerva Tech).

### Isolation of mouse tumors and flow cytometry

The mice were sacrificed and the dorsal tumors were dissected, cut into small pieces, and digested with collagenase (1 mg/ml) for 20 min at 37°C. After the intratumoral injection of OVA_257–264_ peptide, tumor cells were isolated and stained with anti-mouse H-2K^b^ bound to OVA_257–264_ peptide (SIINFEKL)-PE (clone 25-D1.16, BioLegend) or isotype control-PE (MOPC-21, BioLegend). To analyze the local accumulation of antigen-specific CTLs in mice, isolated tumor cells including tumor-infiltrating lymphocytes were stained with H-2K^b^ OVA Tetramer-PE [OVA_257–264_ (SIINFEKL); MBL] for 30 min at room temperature. They were then incubated with anti-mouse CD8-FITC (clone KT15, MBL), anti-mouse PD-1-PE-Cy7 (clone 29F.1A12, BioLegend), anti-mouse CTLA-4-APC (clone UC10-4B, BioLegend), or anti-mouse LAG-3-PerCP-Cy5.5 (clone RTK2071, BioLegend) for 20 min at 4°C.

### Quantitative real-time PCR

The tumors implanted into mice were dissected. Total RNA was isolated from homogenized tumors using RNeasy mini kit (Qiagen) according to the manufacturer’s instructions. First-strand complementary deoxyribonucleic acid (cDNA) was synthesized using a PrimeScript^®^ II first-strand cDNA Synthesis kit (Takara). Quantitative real-time PCR was then performed on an Applied Biosystems 7500 FAST Real-time PCR system using Power SYBR^®^ Green (Applied Biosystems). We assessed the expression of the chemokines CXCL10, CXCL12, and CCL3, and compared them to *β-actin*. Data ware analyzed using delta-delta CT methods. Primer sequences of the chemokines were as described (25), and were purchased from Sigma Genosys.

### Statistical analysis

All statistical analyses were performed using PASW Statistics software, version 18.0 (SPSS Inc.). Statistical significance was defined as a value of P<0.05 based on a two-tailed test.

## Results

### PD-1 expression ex vivo in GPC3 peptide-specific CTLs after vaccination in patients

**Figure 1:**
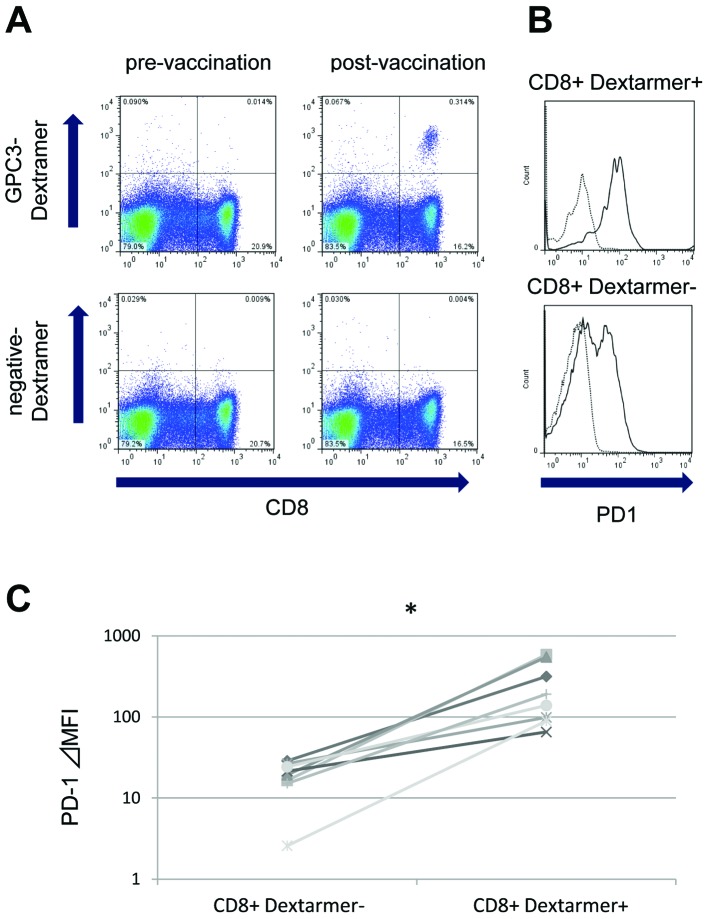
PD-1 expression on GPC3-specific CTLs after vaccination. (A) *Ex vivo* GPC3 Dextramer staining before and after vaccination in a representative case. The frequency of GPC3 peptide-specific CTLs is indicated as the percentage of the Dextramer-positive CTLs among PBMCs. (B) GPC3-specific CTLs were acquired by gating the CD8-positive/GPC3 Dextramer-positive population. The CD8-positive/GPC3 Dextramer-negative population was used as the control. (C) PD-1 expression on GPC3-specific CD8-positive/Dextramer-positive or -negative populations from eight patient specimens. *▽*, MFI, MFI using anti-PD-1 subtracted by that using isotype control. ^*^P<0.05, n=8 using Wilcoxon’s signed-rank test.

To investigate whether vaccine-induced CTLs were affected by the PD-1/PD-L1 pathway, we measured the *ex vivo* expression of PD-1 on vaccine-induced GPC3-specific CTLs using flow cytometry with the GPC3-Dextramer. We used PBMCs obtained from eight patients during clinical trials of the GPC3 peptide vaccine. After vaccination, the frequency of GPC3-specific CTLs increased and could be detected *ex vivo*, as shown in the representative case 1 (Fig. 1A). GPC3-Dextramer-positive CD8 lymphocytes had a higher expression of PD-1 compared with GPC3-Dextramer-negative CD8 lymphocytes (Fig. 1B; representative case 1). In all eight patients with detectable GPC3-specific CTLs *ex vivo* after vaccination, PD-1 expression levels were significantly higher in GPC3-Dextramer-positive CD8 lymphocytes compared with GPC3-Dextramer-negative CD8 lymphocytes (Fig. 1C). Before vaccination, no GPC3-Dextramer-positive CD8 lymphocytes were detected *ex vivo*; therefore, PD-1 expression was not analyzed.

### PD-1 blockade augments the GPC3-specific CTL clones that degranulate against liver cancer cell lines

**Figure 2:**
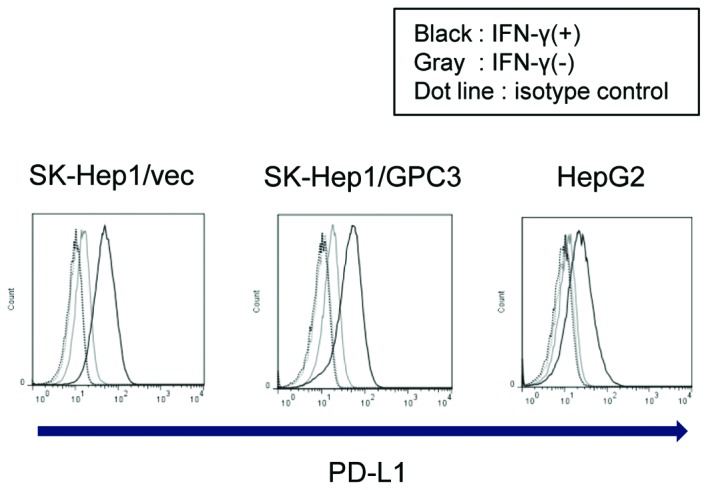
PD-L1 expression in liver cancer cell lines. Liver cancer cell lines were cultured with 100 U/ml IFN-γ for 24 h. PD-L1 expression was then analyzed using flow cytometry. Two independent experiments were performed, which yielded similar results.

**Figure 3:**
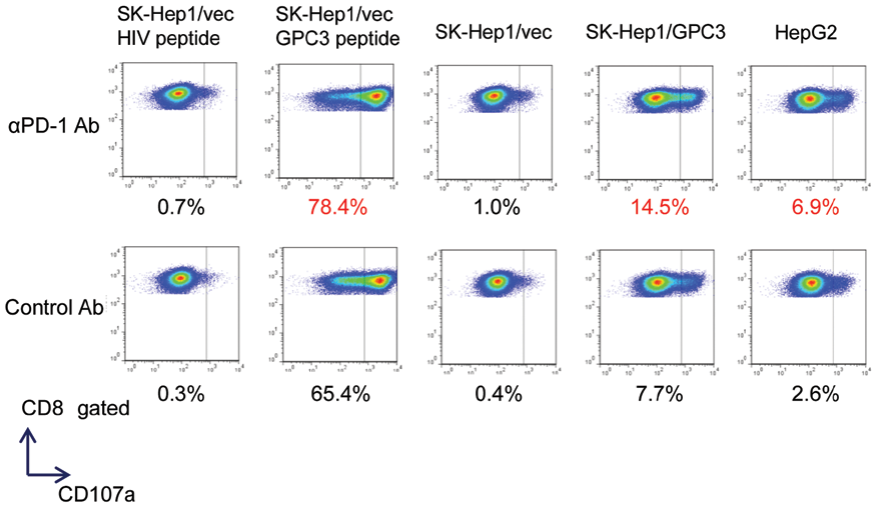
Blocking PD-1 increases GPC3-specific CTL clones that degranulate against liver cancer cell lines. The ratio of GPC3-specific CTL clones that externalized CD107a is shown below each column. The liver cancer cell lines used as the target cell are shown above each column. SK-Hep1/vec (GPC3^−^) cells pulsed with peptide (1 μg/ml) were used as the target cells. The culture conditions are shown in rows. GPC3-specific CTL clones were acquired by gating the CD8-positive population. Two independent experiments were performed, which yielded similar results.

SK-Hep1/vec, SK-Hep1/GPC3, and HepG2 liver cancer cell lines cultured with IFN-γ exhibited marked induction of PD-L1 on their surface (Fig. 2). This suggests that liver cancer cells are invaded by IFN-γ-producing CTLs via the PD-L1-mediated ligation of PD-1. Previously, several GPC3 peptide-specific CTL clones were established from PBMCs isolated from vaccinated patients. These clones exhibited cytotoxic activity against cancer cells expressing GPC3 endogenously (9,26). Therefore, the CD107a (lysosomal-associated membrane protein-1)-mediated externalization of GPC3 peptide-specific CTL clones was examined upon exposure to liver cancer cell lines. The externalization of CD107a could be a surrogate marker to identify the antigen-specific CTLs that degranulate against tumor cells (27). CTL clones mobilized CD107a in response to SK-Hep1/vec pulsed with GPC3_144–152_ peptide, SK-Hep-1/GPC3, and HepG2 (GPC3^+^, HLA-A^*^02:01^+^), but not in response to pulsed SK-Hep1/vec with HIV_19–27_ (Fig. 3). Furthermore, PD-1 blockade enriched the population of GPC3-specific CTLs that degranulated against only GPC3-positive liver cancer cell lines (SK-Hep1/vec pulsed with GPC3_144–152_ peptide, SK-Hep1/GPC3 and HepG2). These results suggest that blocking the interaction between PD-1 and PD-L1 enhanced the antitumor effect of CTLs in liver tumor cells that evade CTLs via PD-L1 expression.

### Combination of a peptide vaccine and αPD-1 Ab suppresses tumor growth in vivo synergistically

**Figure 4:**
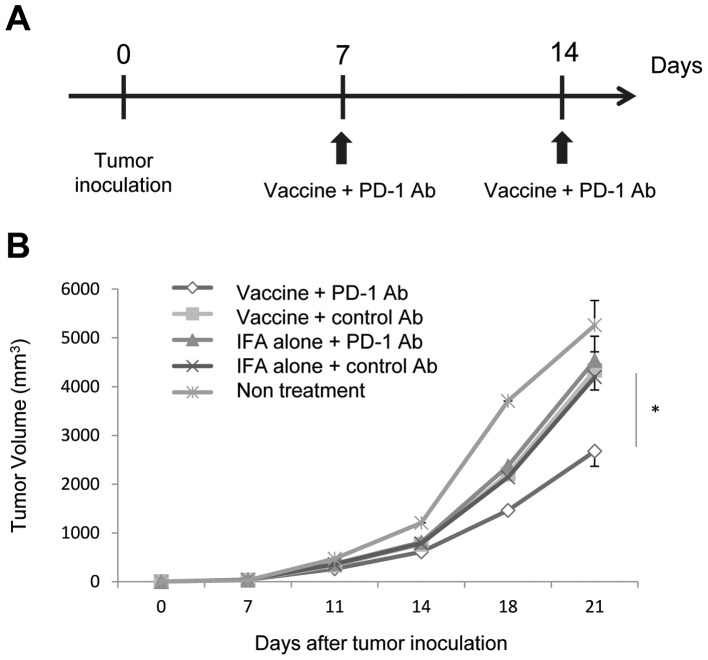
Peptide vaccine and αPD-1 Ab suppress tumor growth synergistically *in vivo*. (A) Mice implanted with RMA were treated with OVA peptide vaccine or IFA alone in combination with αPD-1 Ab or control Ab on days 7 and 14. (B) Tumor growth was expressed as mean tumor volume; bars, SE. Vaccine, OVA peptide emulsified with IFA; IFA alone, vehicle emulsified with IFA. ^*^P<0.05, n=10 using Tukey’s test. Two independent experiments were performed, which yielded similar results.

Intratumoral injection with OVA_257–264_ peptide (SIINFEKL) effectively inhibited the growth of OVA-negative tumors in a mouse model treated with a peptide vaccine (22). Therefore, we performed *in vivo* therapeutic experiments using intratumoral OVA peptide vaccine and αPD-1 Ab in tumor implanted mice. Mice were implanted with RMA tumor cells on day 0, and established tumors (3–6 mm in diameter) were treated with OVA peptide emulsified with IFA (vaccine) or vehicle emulsified with IFA (IFA alone) in combination with αPD-1 Ab or control Ab on day 7. An additional dose of vaccine and αPD-1 Ab was administered on day 14 after tumor inoculation (Fig. 4A). On day 21, one mouse in the untreated group was dead, and all other mice were alive. The tumor volume of mice treated using the combination therapy of vaccine and αPD-1 Ab was significantly less than those treated with the appropriate control (Fig. 4B, n=10). Treatment with vaccine/control Ab or IFA alone/αPD-1 Ab did not inhibit tumor growth compared with IFA alone/control Ab treatment. These data suggest that the combination of peptide vaccine and αPD-1 Ab had a synergistic antitumor effect.

### Vaccine and αPD-1 Ab treatment increases the number of peptide-specific CTLs within mouse tumors

**Figure 5:**
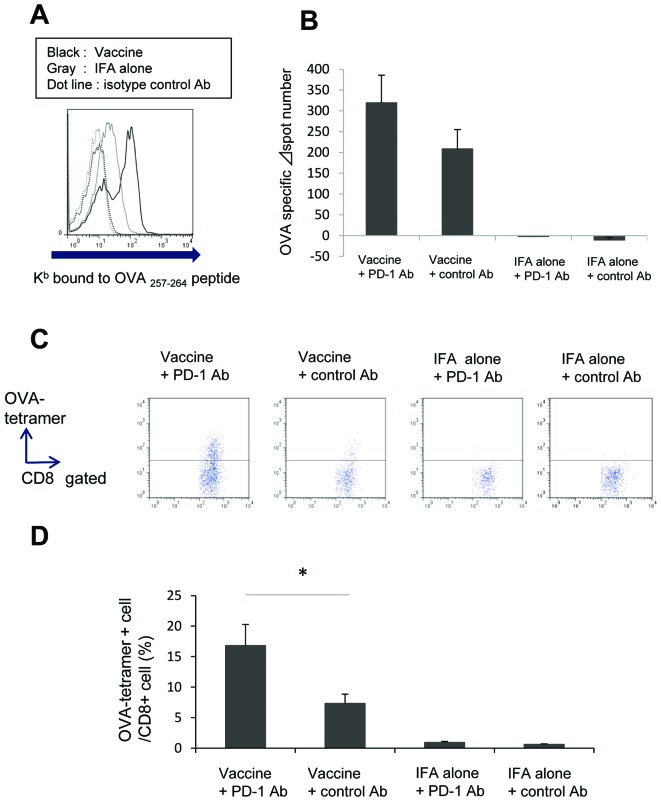
Blocking PD-1 enhanced the infiltration of vaccine-induced CTLs into the tumor. (A) Four days after the intratumoral injection with OVA_257–264_ peptide, isolated RMA tumor cells were stained with anti-mouse H-2K^b^ bound to OVA_257–264_ peptide or isotype control and analyzed using flow cytometry. Data are presented from a single representative sample (n=3). (B) RMA-bearing mice were treated with OVA peptide vaccine or IFA alone in combination with αPD-1 Ab or control Ab. Spleen cells from treated mice were analyzed using an *ex vivo* IFN-γ ELISPOT assay on day 14. OVA-specific *▽*spot number, spot number of OVA_257–264_ peptide pulsed BM-DC subtracted by non-pulsed BMDC. Data are presented as means ± SEM (n=10). (C) Tumor-infiltrating T lymphocytes were analyzed using flow cytometry on day 21. Representative plots of OVA tetramer-positive, CD8-positive TILs in the tumors treated with the combination therapy of intratumoral OVA peptide injection and αPD-1 Ab. (D) The percentages of OVA tetramer-positive cells in CD8-positive TILs are shown from three independent experiments using 2–3 mice per group. Data are presented as means ± SEM. ^*^P<0.05, n=8 using Student’s t-test.

The loading of injected peptide onto major histocompatibility complex (MHC) class I molecules in tumor cells *in vivo* was reported previously using IFN-γ ELISPOT assays (22). In the present study, RMA (OVA-, H-2K^b^) tumor cells were inoculated onto the backs of C57/BL6 mice. When the tumor diameter reached 3–6 mm, 50 μg H-2K^b^-restricted OVA_257–264_ peptide was injected into the tumor. After 96 h, the tumors were dissected, cut into small pieces, and digested using collagenase. To investigate whether the injected peptide was loaded onto the MHC class I molecules in the tumor cells in a solid mass, flow cytometry using anti-mouse H-2K^b^ bound to OVA_257–264_ peptide was performed. The loading of H-2K^b^-restricted OVA_257–264_ peptide onto MHC class I of tumor cells was detected (Fig. 5A).

To evaluate the immunological response to intratumoral OVA peptide vaccine and αPD-1 Ab, the spleens and tumors of mice treated with the same schedule were analyzed as described previously (Fig. 4A). Peptide-specific immune responses were detected in the spleens of mice treated with intratumoral OVA peptide injection using IFN-γ ELISPOT assays (Fig. 5B). Mice that received the combination of intratumoral OVA peptide injection and αPD-1 Ab exhibited an increased number of OVA peptide-specific CTLs compared with those treated with control Ab on day 14 (n=10).

To obtain direct evidence that the combination of peptide vaccine and αPD-1 Ab led to the local accumulation of antigen-specific CTLs, an OVA tetramer assay was performed in mice. OVA-tetramer-positive CD8 lymphocytes could be detected within a tumor using flow cytometry on day 21. Mice that received the combination of OVA peptide vaccine and αPD-1 Ab had a significantly increased number of OVA peptide-specific CTLs compared with those treated with control Ab (Fig. 5C and D; n=8).

### Inhibitory receptors on tumor-infiltrating T lymphocytes and the expression of chemokines

**Figure 6:**
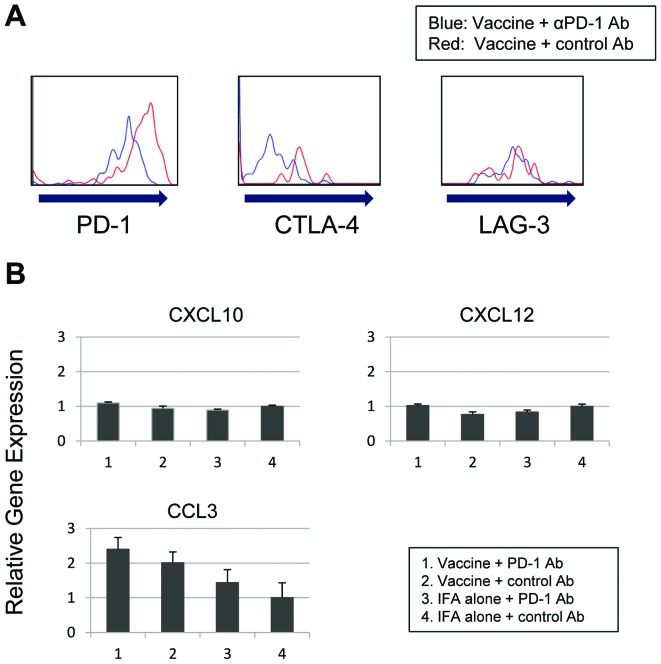
Changes in the expression of inhibitory receptors on tumor-infiltrating T lymphocytes and chemokines at the site of tumors treated using the combination therapy with peptide vaccine and αPD-1 Ab. RMA-bearing mice were treated with intratumoral OVA peptide injection combined with αPD-1 Ab or control Ab. On day 21, mice were sacrificed and the tumors were isolated. (A) Histogram showing the expression of the inhibitory receptors PD-1, CTLA-4, and LAG-3 in OVA tetramer-positive CD8 lymphocytes in tumors from mice treated with intratumoral OVA peptide injection and αPD-1 Ab, as well as from mice treated with intratumoral OVA peptide injection and control Ab. Data are from a single representative sample (n=4–6). (B) The expression levels of chemokines in the tumor were analyzed using quantitative real-time PCR (n=3). Relative expression levels in tumors treated with IFA alone and control Ab were calculated as the control. Data are presented as means ± SEM. Two independent experiments were performed, which yielded similar results.

The expression of inhibitory receptors on peptide-specific CTLs at the tumor site was assessed to investigate the mechanism of CTL accumulation in the tumors of mice treated with the combination therapy of peptide vaccine and αPD-1 Ab. RMA-bearing mice were treated with intratumoral OVA peptide injection combined with αPD-1 Ab or control Ab, as described previously (Fig. 4A). The expression of PD-1, CTLA-4, and LAG-3 in OVA tetramer-positive CD8 lymphocytes within the tumor on day 21 was analyzed using flow cytometry. The expression of the inhibitory receptors PD-1 and CTLA-4 was decreased in OVA-tetramer positive CD8 lymphocytes in the αPD-1 Ab group compared with the control Ab group (Fig. 6A). However, αPD-1 Ab treatment did not decrease LAG-3 expression in OVA tetramer-positive CD8 lymphocytes.

The expression of chemokines within the tumor on day 21 was examined using quantitative real-time PCR. The expression of the chemokine CCL3 was elevated in mice treated with the combination of intratumoral OVA peptide injection and αPD-1 Ab (Fig. 6B). The expression of the chemokines CXCL10 and CXCL12 was unchanged.

## Discussion

Many tumor antigens have been identified in HCC, and their potential clinical utility for the development of cancer-specific immunotherapy has been investigated (28–31). GPC3 is a promising target of antigen-specific immunotherapy because it is overexpressed specifically in human HCC (3,4). In addition, it promotes tumor growth by stimulating canonical Wnt signaling (32) or the Hippo pathway (33). A phase I clinical trial of a GPC3-derived peptide vaccine in patients with advanced HCC showed that it had the potential to improve overall survival, which was associated with vaccine-induced CTLs (8). However, the antitumor effects of the peptide-based tumor vaccine alone were not satisfactory in patients with advanced HCC (8,29–31). Several studies identified molecules associated with the tumor escape mechanism, such as PD-1/PD-L1, Fas/ FasL, and Decoy receptor 3, which might explain the poor immunogenicity and limitations of the antitumor effects of cancer vaccines alone in patients with advanced HCC (16,17,34,35). Therefore, the present study examined whether blocking PD-1/PD-L enhanced the antitumor effects of peptide vaccines in HCC.

The inhibitory receptor PD-1, was upregulated in GPC3-specific CTLs of HCC patients vaccinated using GPC3 peptide, consistent with previous reports of melanoma vaccine trials (21,27). CTLs for some tumor antigens might not be detected directly *ex vivo*. The *ex vivo* analysis of antigen-specific CTLs from uncultured PBMCs could provide strong and novel immunological evidence in HCC vaccine trials. Fourcade *et al* reported that the upregulation of PD-1 and Tim-3 on CTLs was correlated with the expansion of melanoma-peptide vaccine-induced NY-ESO-1-specific CTLs (21). Further studies are necessary to understand the potential clinical efficacy of vaccine-induced CTLs.

In this experimental model, IFN-γ induced PD-L1 expression in liver cancer cell lines. It was also demonstrated that blocking PD-1 increased the number of GPC3-specific CTL clones that degranulate against these liver cancer cell lines *in vitro*. These results suggest that blocking the interaction between PD-1 and PD-L1 enhanced the antitumor effects of CTL in liver cancer cells that evaded CTLs by expressing PD-L1. In contrast, Xu *et al* reported that αPD-L1 or αCTLA-4 Abs did not enhance cytokine secretion and the proliferation of peripheral GPC3-specific CD8^+^ T-cell from HCC patients significantly (36). Differences in the effects of blocking PD-1 and PD-L1 might account for the differences between spontaneous GPC3-specific CTLs and vaccine-induced CTLs.

The combination of a peptide vaccine with αPD-1 Ab enhanced tumor suppression and antigen-specific T cell infiltration into the tumors of mouse models. The exact mechanisms by which CTLs accumulate into tumors by blocking PD-1 are unclear. A previous study in a mouse model of adoptive cell transfer demonstrated that blocking PD-1 increased the production of CXCL10 by bone marrow-derived myeloid cells, which enhanced the recruitment of CTLs in the tumor (25). We hypothesize that the αPD-1 Ab affected chemokine expression, which resulted in recruitment of vaccine-induced CTLs to the tumor. In the present study, the experimental model did not show a change in the expression of CXCL10. However, the expression of CCL3 was elevated by the combination treatment with vaccine and αPD-1 Ab. Furthermore, blocking PD-1 decreased the expression of inhibitory receptors in peptide-specific CTLs at the tumor site. Recently, mouse models revealed that peptide/IFA vaccination increased the antigen-driven expression of the inhibitory receptors PD-1, LAG-3, CTLA-4, and Tim-3 in CTLs, suggesting partial exhaustion (37). PD-1 blockade might be a rational strategy that could be used to rescue CTLs in a state of exhaustion. Interestingly, αPD-1 Ab therapy did not decrease LAG-3 expression in TILs; however, CTLA-4 expression was decreased, suggesting the partial rescue of CTL from exhaustion. A previous study reported that dual treatment with αLAG-3 and αPD-1 Ab was effective in mice with established tumors (38) as well as during the *in vitro* expansion of human NY-ESO-1-specific CTLs (39). Furthermore, Sierro *et al* reported that blocking both PD-1 and PD-L1 might further enhance the antitumor effects of tumor vaccines in mouse models (40).

Based on the results of this clinical trial, the GPC3 peptide vaccine has fewer side effects due to its antigen specificity (8). Enhancing GPC3 peptide vaccine therapy is considered to be promising in terms of sustained tumor control in HCC patients. These data suggest that use of αPD-1 Ab could enhance the antitumor effects of a peptide vaccine, and provide the foundation for the clinical development of a combination therapy.

## Abbreviations

HCC: hepatocellular carcinoma
CTL: cytotoxic T lymphocyte
GPC3: glypican-3
PD-1: programmed death-1
PBMC: peripheral blood mononuclear cell
HLA: human leukocyte antigen
IFN-γ: interferon-γ
MHC: major histocompatibility complex
